# Supplementary material for: Qualitative assessment of healthy volunteer experience receiving subcutaneous infusions of high-dose benzathine penicillin G (SCIP) provides insights into design of late phase clinical studies
Source: PLoS One. 2023 Apr 27;18(4):e0285037. doi: 10.1371/journal.pone.0285037 (PMC10138475; doi:10.1371/journal.pone.0285037)
Supplement: S1 File — (DOCX) [file pone.0285037.s001.docx]

**S1 File: A summary of qualitative themes and supporting quotes.**

The twenty-four participants of the SCIP study were interviewed at four timepoints using a standardised guide regarding their experiences as a trial participant; immediately prior to their infusion, during their infusion, two-hours post-infusion and approximately 7 days after dosing day. Where necessary, timepoints one and two were combined as staffing capabilities were constrained on dosing day, so both sets of questions were asked at the same period. No participants missed an opportunity to be interviewed at timepoints two, three or four. Ninety-two recordings were made and transcribed.

[1 Past participation in clinical trials and reasons for participation](#_Toc129596071)

[1.1 Financial reasons, benefits of renumeration and convenience](#_Toc129596072)

[1.2 Willingness to ‘do their part’ and interest in the research](#_Toc129596073)

[1.3 Unable to partake in COVID-19 trials](#_Toc129596074)

[2 Self-reported pain tolerance](#_Toc129596075)

[3 The consent process](#_Toc129596076)

[3.1 While mostly appropriate, improvements may be required in the consent process.](#_Toc129596077)

[3.2 Participants actively sought out further information](#_Toc129596078)

[3.3 Consent was not sought at every point](#_Toc129596079)

[3.4 Participants expected a needle, not an infusion](#_Toc129596080)

[4 Experience of the dried blood spot procedure](#_Toc129596081)

[4.1 Little or no pain](#_Toc129596082)

[4.2 Painful due to repeated attempts or inexperienced staff](#_Toc129596083)

[4.3 Unsure about what to expect heightened the pain response](#_Toc129596084)

[5 Insertion of the abdominal catheter](#_Toc129596085)

[5.1 No pain experienced during catheter insertion](#_Toc129596086)

[5.2 Catheter insertion described as ‘sharp’ or a ‘scratch’](#_Toc129596087)

[5.3 Catheter insertion was not painful especially when compared to the arm cannula](#_Toc129596088)

[5.4 Catheter insertion was painful](#_Toc129596089)

[6 Experience of the infusion](#_Toc129596090)

[6.1 Pain was experienced by participants during the trial/infusion](#_Toc129596091)

[6.1.1 Specific descriptors of pain](#_Toc129596092)

[6.1.1.1 ‘Stinging’](#_Toc129596093)

[6.1.1.2 ‘Sharp’ or ‘prickly’](#_Toc129596094)

[6.1.1.3 ‘Pressure’ and or ‘heaviness’](#_Toc129596095)

[6.1.1.4 ‘Felt warm’](#_Toc129596096)

[6.1.1.5 ‘Radiating’ or ‘spreading’](#_Toc129596097)

[6.1.1.6 ‘Burning’](#_Toc129596098)

[6.1.1.7 ‘Throbbing’](#_Toc129596099)

[6.1.1.8 ‘Dull’](#_Toc129596100)

[6.1.2 Location of the pain experienced by participants](#_Toc129596101)

[6.1.3 Provision of quantitative pain scores alongside descriptors](#_Toc129596102)

[6.2 Pain or discomfort were not felt during the infusion](#_Toc129596103)

[6.3 Participants felt no discomfort but could feel the infusion occurring](#_Toc129596104)

[6.4 Participants experienced more pain than they expected](#_Toc129596105)

[6.5 Participants experienced anxiety during their infusion](#_Toc129596106)

[6.6 Appropriateness of positioning and privacy](#_Toc129596107)

[6.7 Acceptability of the timing of the infusion](#_Toc129596108)

[7 Experiences in the days and week following the infusion](#_Toc129596109)

[7.1 Pain was experienced but was minor](#_Toc129596110)

[7.2 Difficulties with day-to-day activities or movement](#_Toc129596111)

[7.3 Irritation and bruising experienced post-infusion](#_Toc129596112)

[7.4 Non-specific sensitivity](#_Toc129596113)

[7.5 Little to no effect post-infusion](#_Toc129596114)

[8 Comparison to intramuscular BPG injections](#_Toc129596115)

[8.1 IM BPG was preferable to the SCIP process](#_Toc129596116)

[8.2 The SCIP process was preferable to the IM BPG injection](#_Toc129596117)

[9 Improving the infusion process to ensure acceptability for children](#_Toc129596118)

[9.1 Using entertainment or distractions during the infusion](#_Toc129596119)

[9.2 Provision of additional analgesia during and after the infusion](#_Toc129596120)

[9.3 Slowing down the infusion time](#_Toc129596121)

[9.4 Ensuring information is appropriate for a child to understand and consent to](#_Toc129596122)

[9.5 Using numbing cream prior to inserting the catheter](#_Toc129596123)

[9.6 Using two infusion pumps as opposed to one](#_Toc129596124)

[10 The pain experienced during the infusion was likely too intense for a child](#_Toc129596125)

[11 Overall experience with the trail](#_Toc129596126)

[11.1 A positive trial experience](#_Toc129596127)

[11.2 Participants felt supported by the team and had positive interactions](#_Toc129596128)

[11.3 The trial was well organised and suited participants other commitments](#_Toc129596129)

[11.4 Information was provided often and questions answered](#_Toc129596130)

[11.5 Likelihood of future participation](#_Toc129596131)

These themes will be explored in further detail below substantiated with quotes where appropriate.

# Past participation in clinical trials and reasons for participation

Of those who answered this question, most participants had no prior participation in clinical trials, with the SCIP Study being their first. Reasons for participation can be summarised by the following sub-themes supported by quotes.

## Financial reasons, benefits of renumeration and convenience

Eight people stated that they were driven to participate due to financial reasons and the promise of renumeration. Furthermore, as the study required only one day of confinement it was perceived as more convenient when compared against those with upwards of a week on site. Interestingly at this point in the study (infusion day) no participant deemed the extensive follow-up schedule to be a possible hindrance or inconvenience. Other participants appeared to be genuinely interested in the research and then even more allured by the promise of renumeration. All quotes below are from separate participants.

I saw an ad, like student edge texted me and then they said ‘oh you can get some money and like do a trial at the same time’. So just do it. I was interested and my brother is doing a trial as well and he was telling me about it. I am like why not.

I was having trouble with my car so I figured it might be a good idea to save up some money as well, you know, for the fix. And then I thought to myself like it is also a good idea to get to know what kind of medical research is being done since I am to become like a future nurse. You know, it might be good knowledge to have.

Make a little bit of money and help out, why not.

She (a friend) said you have some spare time why don’t you do this medical research, I said yes as long as it is not anything that is risky.

For starters, it is for one day. It happened to be on a Friday that I could take off…mostly because it is one day.

Something a bit different I guess, and one of my friends recommended it. She stayed like six days. I thought that twelve hours might suit me a bit better.

## Willingness to ‘do their part’ and interest in the research

It was positive that the ‘brand’ of Telethon Kids Institute appeared to be perceived as high among the participants who were motivated to take part in the Trial to ‘do their part.’ All quotes below are from separate participants. Participants appeared to take pride from their participation, and as highlighted by one; *‘I feel good because I am helping.’*

Yeah, because the sponsor is Telethon Kids so, yeah, it interested me. So I do my part.

So I thought to myself maybe you know what it is my work being a part of trying to at least like make other children easier to get a kind of injection without giving them too much pain sort of thing, so yeah.

I would say so because it really goes hand in hand with what I am going to do as a nurse in the future. So if I can, you know, maybe learn something I can maybe apply it throughout my career.

This one it seems to be really interesting because it is for the kids.

Just learning something new I suppose. Help people.

Look, when I was young, I used to do a lot of volunteer work and was always involved in the community. Now the energy levels are not the same to be continuously active..ad now I decided to do something like this, something meaningful, you know. Something helpful and that is not going to be an everyday thing for a long period of time. But I really want to do something nice.

No, no. But that is the reason why I volunteered. That is the first thought that came to my mind is children. They deserve a good life. I see some of the children because I participated in the Telethon for a number of years as a volunteer and it really had an impact on me. I met a lot of wonderful people.

Yeah, I just have a passion for helping sick children.

## Unable to partake in COVID-19 trials

One participant who was particularly keen to be a COVID-19 trial participant was ineligible for those run at Linear so was hence directed to the SCIP Study instead. The emphasis on medical research and the need for trail participants in the midst of a pandemic may have increased traffic to the Linear site; however given difficulties the study had in recruiting the necessary number of participants, this is unclear.

To be honest, because I was really interested in participating COVID trials I hope because we use in our facility we have TEM transmission electron microscope so I studied some because we got some samples and we studied COVID samples as well. I wanted to do something and this was one of the things where, because I know nothing about trial. This is just like.

# Self-reported pain tolerance

There appeared to be a great variation in pain tolerance self-reported by participants. This was not a question that was on the interview guide to be asked of all, but often arose organically as the study progressed. For those who reported having a low tolerance to pain, they often stated to being anxious about what to expect and upon reflection, this may have increased the discomfort they experienced during the infusion process, for example;

I have a very low pain tolerance so that is the only thing that I am worried about but other than that.

Frequently those who had had a more uncomfortable infusion process identified no prior experience with what we described as ‘intolerable pain’, but it cannot be inferred if this was a contributor with their perception of the pain or discomfort felt as part of the study. Most participants who described their pain tolerance did so while reflecting upon the study as a whole and these quotes allow for the most value and insight, provided below.

I think I would be more comfortable if it was okay for you guys to give me more anaesthesia and I did get some more anaesthesia. Because personally I just have a low pain tolerance anyway. So it would have been more comfortable for me throughout the process but again if giving me more anaesthesia will affect the results somehow then it is not really needed.

Honestly speaking, I can manage with this amount but then again I would say, I have I guess a fairly high tolerance for this sort of stuff.

During the infusion when asked for a quantitative pain score: I feel like saying 6 I might even be over rating it, but I am just saying 6 because I normally don’t have any pain at all.

# The consent process

Overall, participants appeared to be well informed about the study and therefore appropriately consented. However, some discrepancies did emerge.

## While mostly appropriate, improvements may be required in the consent process.

Participants reported on many occasions that while the consent process was adequate, there were still elements of the trial that were unexpected. Those that stated this did however recognise that being a trial, this is to be anticipated, but these considerations can improve participant information as the study proceeds to SCIP II. As one participant noted.

From the consent, so I had a rough idea, how it will be done on me. But nothing beats the real thing on the day. So on paper, and on the day, is a little different. Just like the day when the liquid is too dense so it took quite some time to squeeze all the liquid into the tummy. So that part is not being stated in the info and consent….

I think that one is because of maybe, I don’t know but, you can’t really predict what is going to happen when things are going to get injected into you..

Another participant was not aware pain was a likely experience of the Trial but received appropriate information and choices on dosing day.

Interviewer: Had anyone mentioned pain to you before?

Response: No just when I came in

Interviewer: Did they give you an opportunity to opt out at that point?

Response: Yes.

Others wanted more information on the pain likely to be experienced.

…maybe say that you will experience some pain or some discomfort. I was just not prepared.

If I had known that I was going to experience some pain I could have prepared myself better for it but because I thought I wasn’t going to be in pain it just kind of

Interviewer: Caught you of off guard?

Response: Yeah. Caught me off guard.

Other participants conveyed that they were not explained some elements of the trial during the screening process but were put at ease by the Telethon Kids Institute trial staff on the day.

It wasn’t explained too much in the screening process in terms that they were going to take the pictures and stuff, what was it? The ultrasounds. Yeah but when the lady came round, the lady and the gentleman with you, they explained everything that was going to happen. Then I felt pretty comfortable.

I think once we got confirmation that we were going to do the screening, maybe if the screen team could explain that you are going to have some ultrasounds and then maybe just a bit more of the process. What happens? I didn’t really know. I knew that I was getting an injection into the abdominal area but I didn’t know anything else really.

This was not a sentiment shared by everyone, as below:

I understood it kind of like basically. He explained to me how it would under the first layer of fat that they were injecting into instead of like into the muscle and like I could I understand why it could be less painful than a muscular injection. So, I think everything that actually happened on dosing day I figured was going to happen anyway because of how the screening doctor and everyone else on dosing day explained it to me.

Pretty well, so the documentation was there. The person who came around before the injection, can’t remember her name but she kind of talked through it and then prior to the injection, the woman that did it as well, talked through it. There was enough information for me.

Yes, it was pretty good. It mentally prepares a person of what is about to happen. It was good.

From my initial screening and the questions I asked I felt I understood it pretty well especially how it was laid out to me the purpose of the trial. To the point where I was able to explain to other people who asked what I was doing, I think I was able to relate it to them pretty well which means it wasn’t too, I could explain it in lay terms fairly easily.

Everything has been, I felt pretty well informed, pretty confident with what has been happening.

Reasonably well, I got explained to me when they did the physical exam before the dosing and without having an exact, without being an expert in the area I had a pretty small idea of what was happening.

Pretty well. I was informed in advance.

Yes. I was given information about most things and things I had questions on, I was clarified very quickly.

These discrepancies in participant knowledge regarding the Trial highlight the need for a consistent and well-informed screening team and core study group keeping participants and their families up to date as the trial develops and progresses.

## Participants actively sought out further information

Many participants stated to asking extra questions to clarify what was expected of them or to be carried out. In most instances this appeared to be out of general interest but does point to a requirement of all trial staff to continually talk with participants and explain each procedure.

Yes I asked questions the whole time because I was curious as to what was going on, so I understood what was happening.

Me personally I like to know the tools and the ins and outs and the whys of it all, so if I saw a bit of equipment that was being used, I would like to know what it is and how it works. But it wouldn’t scare me otherwise, I didn’t know, but that is just me internally. That is how I like to function. I have got a bit of an interest in medical kind of procedures and things like that. I had done my own semi research before I turned up.

Maybe a bit naïve about it but on the day I was just asking questions. Trying to orientate myself with what was happening. I knew I was getting an injection of penicillin before in my lower abdomen. But some more specific things I wasn’t sure about I just asked on the day.

## Consent was not sought at every point

Concerningly, one participant was not asked if they would allow for extra people to observe the infusion process. This participant was fortunately a very avid scientist, understood the need for further observation and did not feel uncomfortable and while this appears to be a ‘one off’ occasion but has been extracted to reinforce the need to involve the participant in the procedure as an active player.

It is just feedback. Doctor and Joe because you two people and then nurse, these three people should be around, other people we do not want spectators.

Interviewer: Okay. Were you actually asked about other people coming in to observe?

Response: No.

You weren’t?

Response: No.

## Participants expected a needle, not an infusion

Many participants did not understand that the infusion would occur over an extended period of time, instead expecting a needle. For most, this was rectified at the point immediately prior to dosing where the study team explained the procedure in person to the participant.

I thought it was like it went in, so it is like an infusion, not an injection. I thought you just shot it in.

I didn’t actually know it was an infusion, I just thought it was an injection. Like a needle that is what I thought it was but I have never actually had an infusion before.

I didn’t actually know the infusion, I was told it was just an injection under the skin. I was just expecting it to be like a quick injection and then off you go.

As in for the procedure and stuff? Just the infusion, I thought it was just going to be, to be fair though I probably should have put 2 and 2 together when I was told injection and then the volume that was going in. I probably should have put 2 and 2 together myself but it probably could have been explained a bit better.

# Experience of the dried blood spot procedure

On the whole, the dried blood spot procedure was acceptable to participants.

## Little or no pain

Most participants tolerated the dried blood spot well and reported little to no discomfort or pain.

It was just a pinch.

Interviewer: Do you still feel anything from it?

Response: No, it is comfortable.

It was very nice. I mean it wasn’t that painful. It was like a bee sting. Maybe less, way less.

It wasn’t much, it just felt like a pat.

## Painful due to repeated attempts or inexperienced staff

For those participants who experienced a painful first blood spot, it was recalled being due to needing multiple punctures or inappropriate positioning of the lancet reinforcing the need for staff to be appropriately trained prior to conducting this procedure.

They sting me three times in the fingers until they found out the way to do it properly.

Interviewer: So which was better the finger prick or the injection into the vein?

Response: The injection into the vein was faster and easier.

I had no problems with the injection (arm cannula). I think the only problems that people had was with the finger prick test because they had to do it so many times.

It is not the first time, I had it once or probably twice, it was more here. Instead of the side of the finger. I think the area here is harder from here, may be softer, yeah. Don’t compared to that finger prick, yeah that hurt a lot.

Others found the process of actually squeezing the blood from the finger puncture difficult, something that may be able to be improved using therapies prior to attempting to get a sample.

When I first got pricked, of course, you know it is like when you get hit with something sharp but then I didn’t really feel much pain afterwards. It is more the squeezing part. It is not much of a pain when they squeeze it, more from the pressure when they, you know, squeeze your hand sort of thing. Not much of a pain, I guess.

## Unsure about what to expect heightened the pain response

Others reported minor pain only because they did not know what to expect. A countdown was reported as one way of overcoming this, which anecdotally was a method employed by the trial member undertaking follow-ups.

The finger prick was a 2 as well. It just surprised me because it was so quick.

Just like a tiny finger prick. It was more of a shock than anything.

Response: I would much prefer a count down.

Interviewer: Okay, so if we are doing the finger prick, to say like 3, 2, 1?

Response: Yeah. Before the bottom one.

(When asked what pain score the participant would give the dried blood spot): I would say 4.

Interviewer: 4?

Response: (inaudible)..yeah don’t know where it is going to hit.

Interviewer: The anticipation?

Response: Yeah, anticipation.

# Insertion of the abdominal catheter

No topical analgesia was provided prior to insertion of the abdominal catheter and given its placement in an area most people rarely receive injections in, questions regarding tolerability of this procedure were asked. Themes were well defined and are below.

## No pain experienced during catheter insertion

Positively most participants reported very little to no pain during this procedure, with many stating they expected much worse and were pleasantly surprised.

It was all right. I didn’t feel any pain. It was comfortable, it was fine.

The needle was pretty painless. It didn’t hurt at all. But the rest of it kind of hurts.

It was weird because it is not like a normal area to have an injection but it is not that painful.

Response: Okay, that didn’t hurt.

Interviewer: What was that out of ten?

Response: That was like the finger prick.

The insertion was very manageable, I barely felt it.

It was less pain than everything else we had, with the prick and the arm, it was less. Actually, I am quite impressed. I did not feel anything, I don’t know how. Now I am feeling it.

## Catheter insertion described as ‘sharp’ or a ‘scratch’

These words were the most common terms used to describe how the insertion of the catheter felt to the participant. Some participants also reported that this stinging continued as the anaesthetic was inserted but noted that this was described by the trail staff as to be expected.

Just a little bit of stinging, just a sting

I would say it is about the same, the scratch

It was the initial poke, oh that was a shock. It went straight in and it was like the pressure from the anaesthesia, I felt that and then nothing for the first half of the injection nothing and then the second half of the injection was like that, like stinging pain.

Oh it was just a small thing and then the pinch stingy feeling and I guess now is fine

Yeah just the stinging. The pinch and stinging. After that there is nothing.

## Catheter insertion was not painful especially when compared to the arm cannula

A number of participants expressed frustration about the cannula placed in their arms at the start of doing day, stating that it was painful and uncomfortable. Many reflected on that experience when detailing the insertion of their abdominal catheter perceiving it as comparably less painful. Considerations as to how necessary the arm cannula is or if there is an alternate option for placement may be needed prior to SCIP II.

I am not scared of needles but having the catheter in my arm just kind of feels a little bit off and it feels like I shouldn’t be moving it but the nurse told me that I need to move it and use my arm as if it is okay. So I am trying to do that. But other than that, like the pain is not bad.

The needle was probably like 2, but it is not like the catheter is painful it is just really uncomfortable. It just feels weird. It just doesn’t feel like it should be in there.

Similar to the cannula but definitely more subtle. It was just a quick jab.

When she inserted the needle, I found that was way more not pleasant but better than the whole cannula process because I got stabbed four times.

## Catheter insertion was painful

A minority of participants described this procedure as painful, best detailed in their own words below.

I actually felt a bit of pain from the actual needle going in so I don’t know if there is any way to numb the area first… But that was the only thing that made me feel a bit, I had a bit of pain from the actual needle first of all… That was the thing that was out of sort of whack for me, that hurt a little bit, other than that everything else had been fine.

I am not sure. It wasn’t too bad. I felt it more though than the other two (the arm cannula and dried blood spot).

# Experience of the infusion

This was by far the largest theme, likely due to the fact that most of the question guide was developed around exploring this particular experience. There was also great diversity in the pain or discomfort felt by participants with no particular explanation as to why. Anecdotally while the trial team expected those with lower BMI’s to possibly experience more pain, some felt very little or even nothing while those in the higher BMI group experienced significant distress. At present the team are unable to explain *why* some infusions were exceptionally well tolerated while others were not. Quantitative pain scores, as will be discussed, are also not reliable given varied experience with pain and tolerability. This theme has been further broken into sub-themes which will be explored and substantiated by quotes.

## Pain was experienced by participants during the trial/infusion

While it was to be expected that this procedure was not going to be totally pain free (although as will be explored some participants did have an exceptionally uneventful infusion), some participants had a particularly painful experience. In attempting to be as descriptive as possible with this theme, I have further broken it down to demonstrate the words used to categorise or define the pain that was being felt but the following quotes are non-descript and instead demonstrate an overall sense of discomfort experienced during the infusion. The most stand-out description was a ‘step-up’ type pain that many associated with the pump infusing the next millilitre, complicated by the sensitivity of the flow-rate device.

Response: Initially, it was like stinging just from the beginning when they inserted, so when they inserted the needle it didn’t hurt but once they did the anaesthesia and they injected me with it that is when it like..

Interviewer: So the anaesthesia hurt?

Response: It hurt and then it subsided but then it didn’t really stop too much of the pain from happening. It still hurt when the penicillin came and that one was kind of like every time she called out the mls I noticed like that it would hurt again but then it would just kind of like stop hurting and then she would like I clamped at this ml and then it would hurt again, like a little bit more…Like every time she announced the new ml then I would kind of feel it kind of spike a bit and then just subside again. And then she would announce the next ml and then it would spike again.

Participants also expressed that while the initial half or three quarters of the infusion were tolerable and did not hurt, it began to get more intense as the amount left reduced.

Yeah, it didn’t hurt to start off with but then the last five minutes it did really hurt.

I didn’t even feel the like the liquid going in for the first ten minutes and then it was painful.

Maybe, I just noticed that towards, when we got to the bottom half of the syringe, I don’t know what changed but it got noticeably more painful and difficult to do. I don’t know what sort of factors were at play when that happened but, maybe, I noticed you guys fiddling with the knob. I don’t know if you made it go faster or slower but it did

Well the first ten minutes of the injection I didn’t really feel anything but I think after that, the next ten to fifteen minutes it was quite painful. It was like having a stomach ache, I described it like that. But eventually it did go down a bit and then in thirty minutes after the injection, after they removed everything I was back to normal.

### Specific descriptors of pain

More specified descriptors of pain are provided below. For most sub-themes quotes speak for themselves and these are provided in order of the number of references supporting the descriptor (highest to lowest).

#### ‘Stinging’

Approximately half of participants used the terms ‘sting’ or ‘stinging’ with reference to their infusion experience.

The numbing agent when it went in hurt and then it stopped. But then now that the penicillin is coming in I can still feel it a little bit, like stinging.

So it was just like very stingy. It is not like it was burning per se. But say you touched something hot really quickly and then you take your hand off it then it kind of stings like that kind of pain. And then afterwards I noticed for probably like ten or fifteen minutes it still was a little bit, like it felt kind of bruised but like the bruise is really really tiny. It doesn’t hurt anymore. Sorry, it has stopped now.

I didn’t feel much but then I guess towards the end of the dose it was a bit stingy.

Oh, my tummy. It is stinging.

I started feeling the sting really quick and it started to like spread out gradually. It got quite painful afterward, then it goes up and down, up and down, and eventually it just kind of goes.

I just like equate it to like a sting.

#### ‘Sharp’ or ‘prickly’

Another common descriptor were the terms ‘sharp’ or ‘prickly’ which half the participants employed to describe their infusion.

It is a sharp thing, because it is focussed at a point. It is sharp. It is not spreading, it is sharp.

When he was doing the infusion, it was only like a sharp line discomfort was going on because of obvious reasons, apart from that it was okay.

When I breathe it is sharp.

Oh, I feel it there. You know, when they prickle but it remaining and it is increasing.

It is prickly, prickly. Like when they put the needle in initially

But knowing my body, it feels like I prickle myself and then later you start feeling the effects of that light pain, oh, I prickle myself here. You know, for example, prickly pears. You prickle yourself and then you feel something, but then after you feel that something was there. That is the way I feel now. I think it is just the skin, I don’t know. See how they prickle me on the finger now if I touch it, I feel it.

It is still the prickly feeling. I feel like a needle is going in and out kind of thing. Yeah, I can feel that, ooh, yeah. I can feel that prickly needle, you know.

Sharp. Wait, it is all right now, oh it is sharpening.

My first bit of sharp pain, the same level of pain but it like changes from dull to sharp for half a second.

#### ‘Pressure’ and or ‘heaviness’

Seven participants referred to a ‘pressure’ or ‘heaviness’ being felt, generally as their infusion reached the latter half.

When it was in I felt quite heavy pain.

That sensation is hard, the liquid going in the body.

But it is kind of tender, I feel it. I can feel it yeah. Ooh, I can feel it now. Is he pressuring a little bit, that is what I am feeling.

Then at the two thirds point, then I could start feeling like pressure, like a generalised spread over an area. So it wasn’t like full blown at that point it was more like the pain was pressure that I could feel. It was still painful.

There is now a feeling of pressure, like pushing pressure on my abdomen.

It still feels like pressure. It feels like there is something heavy across here.

It doesn’t sting any more, it is more like the pain has been there a long time, so it is like lingering pain. But it feels heavy, it is like heavy pain.

No, just may be like light pressure, very light pressure.

the first two thirds was easy but the latter part was just like kind of like uncomfortable, like just the pressure from the injection.

#### ‘Felt warm’

A smaller number of participants noted that the area around their infusion site (abdominal) felt warmer.

Talk to me about the heat that you experienced?

Response: Yeah, it was just kind of like, it just felt like it was kind of hot.

Interviewer: Just where the insertion was?

Response: Yeah, where the insertion was.

Interviewer: As a whole your body didn’t feel hot?

Response: No, just in that specific area.

Just the area it feels, there is a warmth where it is happening but it is so subtle. I can feel there is activity.

Yeah, it feels really warm. It feels like it is hot as well.

Now it just feels really hot in that area.

It feels a little bit like warm, that is all I can feel.

An additional subset of participants also felt systemically warmer, which may have been associated with anxiety or excessive pain.

I don’t know if it is the effect of the medicine but maybe a bit warmer.

I am getting warm. Like temperature warm yeah.

Response: I am superhot.

Interviewer: The pain?

Response: Is more like uncomfortable.

It wasn’t too bad. But it is hard to determine whether when I was getting really hot was due to the injection of not but I don’t think I would sit through and have that again. Just from the experience of sitting through it. I don’t think I would want to do it again that is for sure.

I am sweating.

#### ‘Radiating’ or ‘spreading’

Six participants identified a ‘radiating’ pain that was commonly associated with a sharp or stinging sensation.

At first it was kind of like a sharp pain but now it is just like radiating pain, like throbbing almost.

Ow, it is stinging. It is like shooting pain down.

It is like shooting pain. It stopped and then it is shooting down that way.

That is like shooting pain. Why is it only one way?

I started feeling the sting really quick and it started to like spread out gradually. It got quite painful afterward, then it goes up and down, up and down, and eventually it just kind of goes.

#### ‘Burning’

Two participants described the pain they felt around their infusion site as feeling like burning.

It is like it feels like I am getting burnt a little bit. You know, like when you have a fire and you touch the tip of it very quickly. It is just like that kind of stinging.

Yeah, it was like, it feels like burning your skin or something. That is how it feels. But then like when they stop, it gets tolerable. I can stand it now, it is fine. When it starts like getting in, oh, that is it. Now I have pain but it is fine, I can stand it. So it is okay.

#### ‘Throbbing’

Four participants experienced a ‘throbbing’ pain that they associated with a cramp or stomach ache.

At the very moment, it is kind of throbbing.

Interviewer: Okay, and how would you describe that?

Response: Like a cramp, intense cramp.

More like a throbbing

Kind of like a tummy ache, kind of thing.

A slight throb. The pain is quite manageable at the moment.

#### ‘Dull’

This was the final word identified thematically to describe the infusion pain and was used by only two participants.

Is it dull or sharp?

Response: Dull.

It is a really strong dull, occasional sharps… But when the injection hurt, I felt a few sharp pains at the beginning of it then it slowly developed into a duller pain that was quite constant. Halfway through the injection I think based on the injection about hurt a little bit and it did increase. It was quite manageable. Then I felt this bruising lump there, which seems to be common.

### Location of the pain experienced by participants

Attempts were made to determine exactly where participants felt the pain of their infusion and while generally abdominal and subcutaneous, a myriad of descriptions were provided.

The pain starts somewhere underneath the skin.

Yeah, like I can feel it just above my abdominal wall.

Yeah, like one place like just at the injection site, maybe shooting sideways

It definitely felt on top of, like I could feel it on top of like my abdominal muscles and like in between that layer, like subcutaneous fat.

No it is still focussed at there. Now it is getting better. I think it is not getting better but I am getting used to it.

Is it localised?

Response: Yeah, just to that area.

It is like a dull pain not radiating, yeah, it is present. In the one spot.

Yeah, just around an inch or two. Like the circumference.

### Provision of quantitative pain scores alongside descriptors

While a qualitative piece that should err from the provision of quantitative data to support quotes, a number of quotes below that include both a pain score and a descriptor are provided. If anything, this theme should demonstrate the diversity of pain tolerance and experience, specifically individual perception of pain.

I just feel a second spike. Once again I rate syringe insertion from 0 to 10, it was 1, and this thing is like 0.1.

Like once he finished it dropped to like 5 or 6, but now that he touched it is like yeah, it hurts.

Yes. At the end of the injection it was when I experienced the most pain. At the beginning it was really nothing, I didn’t experience severe pain, at the end it was increasing, the maximum I got was like 7, 0 to 10. But it was not a bad thing, it was manageable.

No it is just, it goes up to 8 and then it stays there. It is not dropping.

What is your pain score?

Response: Nothing.

Interviewer: Any change?

Response: No.

I feel like saying 6 I might even be over rating it, but I am just saying 6 because I normally don’t have any pain at all.

Like a 7 for a second. My descriptions are confined not much

Just a bit, still 6, but we are getting in high 6s. Now that I am taking deeper breaths it is getting a little uncomfortable.

It is the same. It is sort of a 3 and 4. I can feel it now. It is increasing. Ooh, now I am feeling it. It is increasing yes. The feeling when you have a needle in your arm, it is increasing a little bit.

What would you rate the pain as?

Response: I would go for a 7 now.

Do you want us to stop?

Response: No. It is tolerable but I am just trying to gauge it to what we started with. Right?

I can feel that, a 10. I can feel it, ooh, yeah. It is a very uncomfortable kind of pain. It is a little bit less now. It is increasing again.

That was like 3 I think. That was like when, a minute after I started feeling it. It started getting really like painful.

The pain, maybe the 6 might have there. I just felt more than before. A bigger number more than before. Not sure if it was a 6 or not, 10 is when you screaming on the floor. I am not sure what that feels like, I just know it is more than before.

## Pain or discomfort were not felt during the infusion

This was a positive theme that emerged from the quotes, particularly when contrasted against those who suffered a physiological response (i.e., sweating) due to their infusion pain levels being so high. Again, it cannot be inferred as to why these participants (approximating ten) had such a tolerable experience. Quotes supporting this theme are also relatively brief – somewhat expected given the infusion went off with no hitch and participants were not inclined to further detail their uneventful procedure.

Everything is good, yeah. All went smoothly. No comment, all good yeah.

No, it was pretty much everything that I expected. Yeah, I was pretty happy.

I mean I didn’t feel any discomfort at all, yeah, but maybe yes some people might feel that but I felt pretty comfortable how I was.

I am pretty happy with everything. I feel that itself was pretty professional and I am pretty happy with how everything was explained.

It doesn’t feel like anything is even happening. The prick that went in that was the most pain that I have had.

I actually thought it would be more painful that it was but it wasn’t too bad.

Painless yeah, really easy.

Yeah. It was perfect. I don’t know if it makes a difference, longer or shorter. Yeah, it was good enough.

Given the infusions occurred in a hospital-ward style room with only curtains for privacy, it was difficult to keep participants unaware about the experience others were experiencing. It is likely that this may have heightened anxiety immediately prior to the infusion, especially for those who were beside someone who had an intolerable infusion and is a consideration for further iterations of this trial.

I wasn’t feeling much pain, but I guess like, from what I have seen with other participants they seem to be much more in pain compared to me

I was surprised at the speed at which it went at, I think my dose only took ten or eleven minutes, I think, and the guy next to me took a bit longer, so that was what I was expecting. I could hear obviously through the curtain. So I was expecting that and then when I was told I only had few minutes left.

Others reported feeling at ease by the trial staff and the amount of activity that was occurring around them. For most, five members of staff were present; the qualitative interviewer, the radiologist, the doctor running the infusion, the trial PhD student and a Linear nurse.

You were asking me, so like putting me at ease but also taking my mind off the fact that I am meant to have a needle jabbed into me and then the dose. So it just kind of took my mind off it. I was never alarmed or concerned at all through the whole period

Lastly, while those who experienced little to no discomfort were pleased with the outcome, they were reflective about how this procedure may be tolerated by a younger person. This will be further explored in proceeding themes.

The discomfort is pretty tolerable. If I said it was 0.5 or about 1, if a younger person said maybe 3 or 4. I don’t think that is serious discomfort. I think that is the normal pain you associate with certain basic procedures.

## Participants felt no discomfort but could feel the infusion occurring

Approximately nine participants, many of whom could have their experience also categorised in the above theme noted that while they felt no discomfort or pain, they could feel ‘something’ nonspecific. This theme has been extracted given trial staff lack the ability to know for themselves exactly what this procedure feels to participants and potentially future patients. Quotes stand alone in expressing this theme.

I feel it, it is there. You get used to it quick, it is like. I cannot explain it. It is not painful.

I don’t know how to describe it. I can’t tell I can just feel it because I know it is happening or like probably feel like the sensation of it.

The biggest problem if anything was the prolonged feeling of having a vaccine needle in because when the fluid was going in I had no, kind of, how much to put through, I was feeling that, the flow. Until it became very steady but at no time except at the end when I had a bit of pain, but everything was very manageable, tolerable. I had no issue with that.

That wasn’t like a pain, it is like sensitive, like I am feeling myself inside. It is not a pain. It is just like, yeah again, if you have a really deep wound, of course you might have a little bit of pain but you can feel everything inside and it is quite sensitive, the same here.

I could feel it. It wasn’t like painful but I could feel it.

You know something is going in but you don’t really feel the pain. Like you are scratching something like that.

Interviewer: Are you feeling anything?

Response: No, I did feel one initial, but you know, and then it has gone in. Not like a pain.

Interviewer: Just movement or pressure?

Response: Yeah, probably just the movement.

Response: Funny how you can see the area.

How does it feel?

Response: I can feel movement.

Painful?

Response: Yeah, kind of like a little heartbeat.

I am definitely feeling that something is going in.

Interviewer: You’re feeling that something is going in?

Response: Something going in yeah.

Interviewer: But not a bad sensation?

Response: A sensation. It might be uncomfortable for others but for me it is fine.

It is very little. I feel like there is fluid going in or something. This is stronger now. I am feeling it now but it is not painful for me. It is the same as the finger prick. If not less. Yeah, I can feel that but it is not major. I wouldn’t even call it pain, it is like I feel something cold, a little bit cold.

## Participants experienced more pain than they expected

A number of participants (six) stated to feeling more pain than was expected. This somewhat links in with the theme about consent but is also a difficult expectation to manage in a phase 1 clinical trial where there are almost no knowns or guarantees about a participant’s experience.

I mentioned how the anaesthesia one kind of like shocked me a bit because I thought it would be painless but then obviously I experienced some pain and that kind of shocked me

To be honest, not really. I guess, not like I felt today. I didn’t expect it to be that painful. I expected it to be painful but not that much.

I do not expect the actual infusion to be as painful as it was but I think from like when I saw the other participants they did not go through as much pain as I did, I guess, so I don’t think the actual doctor expected it to be that tedious.

Maybe, I just noticed that towards, when we got to the bottom half of the syringe, I don’t know what changed but it got noticeably more painful and difficult to do. I don’t know what sort of factors were at play when that happened but, maybe, I noticed you guys fiddling with the knob. I don’t know if you made it go faster or slower but it did.

There was definitely more pain than I expected but it was fine.

## Participants experienced anxiety during their infusion

For a minority of participants, anxiety stemming either prior to the infusion or developing as it continued was a concern and something to consider managing for SCIP II. Ways of managing this include practicing the infusion so unexpected outcomes do not concern participants, distracting participants and ensuring appropriate informed consent.

I was a little bit worried. So yeah man, it can’t get in, so were they going to put it in again or wait. So but after a while they managed to get in. So not too bad… I was a bit worried that is all. Are they going to poke again, I don’t know what is going to happen next.

Interviewer: I just want to explore because, you know, like when they said the mls then you felt the pain come on, you looked distressed. Talk to me about that?

Response: It was just a pain that was coming through, like the stinging and I could also feel her kind of like a little bit lifting it up, like the

Interviewer: The tension?

Response: So I was like, I didn’t want it to come off. I was kind of scared

I had never had an injection into that area before so I was a little bit afraid of how the pain was going to be because we are not familiar with that kind of pain.

## Appropriateness of positioning and privacy

Several participants explicitly stated that they were very comfortable lying down during their infusion and appreciated the privacy that was provided to them, especially considering the location of the procedure. One participant who recalled being slightly elevated propped himself up and it made no effect on the infusion indicating that patient preference should always be a consideration.

Interviewer: Do you think you might have been, were you comfortable lying flat during the process?

Response: Yeah. Elevated is good.

Interviewer: You were a little bit elevated or were you flat?

Response: I remember I think it was elevated.

Interviewer: Was that good for you?

Response: It was comfortable.

Interviewer: Would you have liked to be further up or further down?

Response: It was just nice.

No, I was flat, I was pretty comfortable as it was. Yeah, I was pretty comfortable.

The most uncomfortable part was the anaesthetic and after that I was lying flat and no pillow behind my head, or a very like small one. I wasn’t really able to look or see what was going on and then it was just a matter of waiting. I was comfortable.

No, I just mean like someone doing this, not reacting at all just going oh yeah this is nice. Just sitting there not saying anything.

They pulled the curtains around so I feel that my privacy was dealt with accordingly.

I think lying down for me was good. Because I think sitting upright probably would have hurt more I would be in a crunching position and then the muscle would be like tense, so I don’t think it would a good idea.

## Acceptability of the timing of the infusion

There was great variability in the acceptability of the time the infusion took. For some – notably those who experienced no pain – they felt it could have been shorter. Others thought it could have been prolonged a bit more given their association of pain with infusion speed, while others were accepting of a larger amount of pain over a shorter period. Results in this theme are also complicated given a subset of participants were expecting a needle, not a prolonged infusion and therefore were probably not mentally prepared for twenty minutes of intense observation with a large trial team.

Yeah. It was perfect. I don’t know if it makes a difference, longer or shorter. Yeah, it was good enough.

It was actually very quick. I kind of expected it to be a bit longer.

The time taken, yeah, it is acceptable.

Yes. I guess, it is long enough. I think this can be improved, the procedure, I mean less time. But I think it is you are starting to do it. Yeah it is good.

I think it is one of the most, if it was done quicker.

Interviewer: So a lot of pain very quick instead of a lot of pain over a shorter.

Response: A lot of pain over a shorter period, rather than just pain

See I guess like that length of time wouldn’t be too bad if it didn’t hurt for the full half an hour but could you imagine, like I couldn’t imagine sitting for half an hour with that pain that I had that second time around. Especially being a child like you wouldn’t sit still with it, and if that was under general anaesthetic as well, I would had to know what it would be like without the general, the local yeah.

If you didn’t do it over a time period would it suck. Like if you injected someone with this.

I mean definitely getting the infusion was, I won’t say it was painful but it took a while. I mean because of the amount that I was being dosed with.

Half an hour is painful. Talking did help.

# Experiences in the days and week following the infusion

As participants were interviewed on day 7 post-infusion, a larger picture of the effect the procedure had on their day-to-day life – particularly for those who experienced no pain on dose-day – was enabled. No serious levels of pain were reported and the following themes are substantiated by quote lengthy quotes which allow for a better understanding of how participants tolerated their infusion.

## Pain was experienced but was minor

As aforementioned pain was present for some participants in the week following their infusion but did not appear to be particularly limiting.

I think at the beginning maybe side effects like remaining pain on my skin but everything is going well. It did not affect me at all, physically

It is just tender where I was injected at.

I knew that I might be uncomfortable or feel some kind of like pain around the injection site over the next few days anyway. So I prepared myself for that

Yeah just the discomfort for the first two days and then after that it was fine.

Day one was like that, that was when I had the high amount of swelling from bending over at work and being in a really busy environment. That was probably the day of most concern for me I would say and then after that it was just like discomfort. Learning to work with it… I think the abdomen was a little bit limiting because you use your core for pretty much everything especially like the movements of bending down and bending over and twisting to the left and right. You really feel it for that first couple of days but then I was noticing like I didn’t want people to like get near me because the waist is such a common contact point with people especially in my work place. I was constantly like very aware and moving people away from me.

When I am touching it, I can feel it. If I don’t touch it, it is fine.

I had an idea it the injection point was going to hurt for maybe a day or two but then the size of the actual mass that was there. It wasn’t alarming or concerning. I asked her again today what it was all about. You know, what the body does to make that thing there. Just interesting rather than alarming as to how the body works and why it was just because it was. Hard to touch, over time, now it is reducing. No real restrictions or anything like that I don’t think, or not that I know.

I kind of didn’t find it painful but it definitely was uncomfortable when it is there and stuff.

I think Sunday/Monday was my worst day. I was in the most amount of pain. So Day 2 and 3?

I have had to take Nurofen twice and only when I have just gone to sleep. Probably didn’t even need it, but I did. But it has been good so far.

I mean it wasn’t like I couldn’t do anything but I definitely felt it more and I was getting more shooting pains, sharp pains even just by sitting. Clothes were rubbing on it and it was hurting but I could still function. I feel like I have got quite a high pain threshold so I don’t think it would be the same for everyone. Other people might struggle.

The most challenging, probably just the initial pain after a few days of the injection site, particularly the bruising, not the immediate days after but a few days after that, I would say around I think day 3 or 4 was when the bruising seemed to be at its worst which just meant the pain was at its worst without me doing anything to aggravate it further. Without me bending over or moving much I would still feel that pain. That was probably the most difficult part of the whole thing but that was quite easy to deal with in the grand scheme of things just a bit of bruising. Nothing I hadn’t had before.

I was quite sore from the injection site the first few days but other than that it has cleared up.

From personal point of view, I don’t think I have had an adverse effects. You know, you get a bit of an injection, you are sore for the next couple of days, it is part of getting the injection.

Well actually no it was the second day I just remembered. I popped out to get the paper and then I remember coming back and I sat in front of the TV and I said Whoa, this pain, now it is not even me lying on my side, just lying on my back I am really feeling it. So the second day is when it (inaudible). I don’t know if it is also psychology thing because when I was in the hospital the nurses were very, you know, maybe it just was really registering as much of the pain but the next day when I was left to my own devices.

That night maybe I was starting to feel a bit of tenderness, but really it was the next morning and the day after, those were the two most tender and painful. I had to be the most cautious on those days. The Saturday and the Sunday and maybe the Monday as well.

Day 0 to Day 1, was nothing there, Day 2 it kind of caught up with me. Again not alarming...

It was a bit hard to deal with the next days but it was just a bruise. It wasn’t like it felt like something was broken or didn’t feel like something was like wrong. It just felt like a bruise. So it was manageable.

## Difficulties with day-to-day activities or movement

Due to the pain or discomfort some participants experienced, they noted actively altering their normal activities or moving differently to avoid further irritating the area. This is an important consideration given the current therapy of IM BPG often limits patients in the days following the procedure.

Yeah, yeah, and just like wearing your socks and you bend down and your tummy is in the way. That was the hardest. Other than that, pretty okay.

The following days, probably the first two days were probably the most uncomfortable, but that was in a combination with working as well…Just like the bending over, like doing work activities in like a crowded area. So working in the bar, like a very crowded bar with lots of people around, like I felt like I was almost guarding it sometimes because it was painful and then I noticed the swelling on the first day of working in the bar where I was like bending over heaps and then half way through the shift I stopped bending over, I stood and it reduced in size. Then the following day at work, it was a much quieter day at work where I was really able to minimally bend over and moving and it was a lot better that day. Then on the Monday I was cautious about going to work because my other job is quite labour intensive so like working outdoors with lots of heavy machinery and equipment and I didn’t want to like flare it up again in the morning or on that day so I decided not to go to work that day and then the following four days after that I was predominantly working in like a library so I was sitting down but I was like doing daily activities like walking, cooking and etc.

But it wasn’t that bad, especially the first day when I got the injection. At the end of the injection it was hurting but it wasn’t like crazy pain. That first day I felt like this is very nice nothing is going on but the next two days were like, yeah, I could feel like yes it hurts and I have to do my ways to try to sleep. That was my main limitation, sleeping, and of course, dressing I had to be careful.

Like when I was bending to put my socks on or my shoes, that was like the hardest part because the more I bend the more I felt the pain in my abdominal area. Especially on Sunday was the worst day, Monday it started to get better and by Tuesday it wasn’t a problem at all.

Oh well, going to work was a bit harder, I couldn’t exercise. That was pretty much it. It was just a little, the pain I felt after the dosing that was all, but after that yeah I was fine. Nothing else.

Really that first weekend, the only challenging things were the physical motions of the day interrupting the pain and the tenderness of that. Maybe the colouration was unexpected for me, to see it go from so bruised to then yellow really quickly.

Sleep. It being on a particular side of the body, like your abdominal wall, it felt like the first couple of days you won’t be able to sleep on that side, so you just have to be careful not to roll over to that side. Like if you irritate the site.

Then I had work that night from 10pm to 3am in the morning, and it was quite challenging when I bent down and I tried to pick things up, that was a bit of a challenge. I have had to squat a lot more than I usually do because it feels like it is preventing me from doing certain movement. Overall, it disappeared in two to three days.

## Irritation and bruising experienced post-infusion

This was an element of the trail closely examined at each follow-up visit, hence additional data will be available in the form of photos and trial notes. However, a summary of quotes regarding the bruising or irritation experienced by participants are provided. Positively the bruising experienced did not overwhelmingly concern participants.

Initially there was some foreign body in you, so when they squeeze it in I can feel just a little bit of pain, so initially you can feel something is there but because there is local anaesthetic not much of a feeling, just a little bit but not much. But once it wears off and you start to get a little bit of irritation, a few hours later.

I have not really had too much pain. I had a bit of itching yesterday, more looks than anything.

The one was probably just yesterday the itching and I was just trying to avoid itching it and just getting it out of my mind. Other than that it has been pretty straightforward.

Interviewer: The bruising, the bruise that formed, was that sort of concern?

Response: Not really, no. I mean I understand that when we come to do these trials these things may happen and obviously with training in the future and how to do different things and I understand that so it was not really a concern to me.

It is slightly red…I did feel a bit of a lump, I guess.

The only thing was the swelling for about three days after. But after about three days it did not bother me much.

The bruising I wasn’t too worry about. It just looked visibly bruised but not particularly worrying. If it hadn’t cleared up in like a couple of weeks I would have been worried but it just cleared up so it is just a bruise.

I did notice bruising the next day and got to circling. The most extensive bruise occurred up until the second day but afterwards it started to disappear, now you can’t even see it anymore.

## Non-specific sensitivity

A couple of participants noted that while they did not feel pain, there was a feeling that ‘something’ was present around their infusion site and they were more aware of their abdominal region than usual.

No I think after the anaesthetic because you start feeling a bit more, I don’t know, heavy on the site of application…Yes, the day after I felt more, maybe, kind of faint, again it wasn’t a pain it was just a feeling that I had in my skin. It wasn’t a pain really in my opinion.

It is just a sensation there, it is not like painful sensation, so it just when you have a bruise, something like that, yeah.

## Little to no effect post-infusion

Approximately seven participants identified little to no effects following their infusion – a positive result. Some in this group did experience pain as the days post-infusion followed but none were serious.

There was something there, I felt something but nothing

Okay, so you are not feeling any pain now?

Response: No.

Interviewer: Okay, because that was going to be my question, are you experiencing any discomfort or pain now?

Response: No.

Interviewer: Okay. Did it affect you sort of getting up and going for your meal?

Response: It didn’t really affect me. I think it was just like I was a little bit worried, like if I do something wrong like I might hurt myself but then I realised as I kept going I wasn’t actually hurting. Like it was just a little bit uncomfortable.

I think that is a very good thing because once it finished the injection the pain went down really fast. At the moment I don’t feel anything.

It wasn’t as bad as I thought it was going to be in terms of the pain.

On day 7 I don’t feel pain at all. It has reduced by quite a lot. I feel much better. I hope whatever resource I got from this it is able to prove that the process can go through very smoothly.

It was just a discomfort around the injection site and I felt a bit tired one to two days after but after that I was fine.

# Comparison to intramuscular BPG injections

Three participants had prior experience with intramuscular BPG injections and were therefore able to compare the SCIP process with the standard therapy.

## IM BPG was preferable to the SCIP process

The only participant who stated that the IM injection would be his preferred standard of therapy was also a participant in the intramuscular versus subcutaneous injection study also run by Telethon Kids Institute a few years prior. His SCIP experience however stood out as being particularly uncomfortable for reasons hypothesised to be shallow needle insertion and a poor night’s sleep.

Interviewer: If we had to do this again, what would you like to do different?

Response: I probably would prefer if was (inaudible)

Interviewer: You wanted it quicker?

Response: Yeah.

Interviewer: Okay. So you preferred the IM. That is interesting. You are the only person who is in both studies. Do you reckon this study is appropriate for children?

Response: Oh, no. For me, I feel like as a kid, if I feel that much pain I would probably (inaudible… I wouldn’t do it again. Probably no.

In a later interview (Day 7) this participant stated that knowing the shallow needle was likely the reason for the pain they experienced, they would be willing to partake in a repeat of the study.

Interviewer: Would you do it again, if I said I would put it in deeper, would you do it again in say like three- or four-months’ time?

Response: yeah. (inaudible) I do trust you so I absolutely yeah I will yeah.

## The SCIP process was preferable to the IM BPG injection

Of the two participants who perceived the SCIP process to be less painful and an easier process, one was able to distinctly recall their IM experience and therefore provided a very detailed comparison over multiple timepoints.

Mid-infusion

You know, I recall when you have an injection in your bottom, right. They inject it that is what I feel.

Interviewer: Well that is a good descriptor because this is what we are meant to be comparing it to.

Response (mid-infusion): It is increasing. That is okay. But it is tolerable for me. What is the one that really hurts that they put in your bottom when you have pneumonia.

Based on what I can remember I would prefer this.

2 hours post-infusion

Yes. The other one is painful really that I remember but this one is kind of, if I touch it, it is a bit painful but otherwise, from what I remember, I couldn’t even walk because of the pain. But with this, what you guys did this morning to me I think it gets you back to normal if you will right away kind of thing.

7-days after infusion

Yeah. That injection I can remember it clearly, vividly. I felt like I was limping after because I really felt it, I think they do it in the muscle or something. So it was felt for a day or two, if not three. But with this one it was different. You had that feeling but that pain and discomfort did not remain for long.

When asked, the other participant who was able to recall a comparison simply stated *‘this process is better.’*

# Improving the infusion process to ensure acceptability for children

Participants were asked specifically how the infusion process could be better tailored to children, with considerations to how their own experience was and elements that may have made it more tolerable. These quotes are very detailed and helpful hence have been provided in full despite their lack of brevity in many instances.

## Using entertainment or distractions during the infusion

By far most participants noted distractions as being critical to ensuring a successful infusion if it was to be undertaken on a child. This included games, music, blankets or a support person.

Maybe they need distractions, maybe some TV or some games or something to take their attention away from the injection.

Something for them to play with so they are not so occupied with looking at the injection area.

Yeah, I think like maybe as before, when giving the injection to like distract them so as not to make them so worried or to take their mind away from what is going to happen so that they will try to ease their pain. So I think there is nothing much that you can do because a needle is a needle.

Yeah, it would be good to have someone around.

Yeah, a distraction would be good for children. You know, like if they get vaccines they play with the kids and like pinch them around so they don’t feel where the actual needle is going. I think for them yeah, so that they don’t see it and they don’t really feel it and they are distracted but apart from that I can’t really say anything that would improve this.

I think for the most part it is just like distracting them from it happening. Giving them something else to do or focus on instead of like just having them lie there and look at themselves getting an infusion.

One can be feeling a little bit of discomfort but apart from that it is a good idea when someone is around, apart from the doctor who is chatting, you know, because it took some time for him to insert the drug in my body so one can get anxious about it. Let the doctor do his job, and the doctor is doing his own part, if someone is around here like in your position. Just it is a good idea to distract the people, the young one’s mind, whereas a guy of my age they understand things you know.

Especially if you are a kid, you need to feel distracted or something. When I was a kid I was really afraid of needles but it helped to grab something. I used to grab my Mum.

If I think like a child, even if he doesn’t feel any pain, he is going to feel really scared and grabbing something definitely helps with pain and to focus not to move. So I would add something to grab.

Maybe a personal toy, that they like or Mum hands. That would be nice.

Something to hold, maybe it can be like a stress ball definitely it would help. That would be nice. Yes, I would have a stress ball.

Especially if it is a small kid that will distract them from the pain also to help us to focus on something not in the area getting affected.

Oh yeah, maybe, some kind of distraction. TV, and I guess their parents would be in here with them anyway,

Because I guess if you are distracted, like you distract the kids with TV or something they might, because they are pretty resilient. They will probably feel something then be distracted by TV or something. Yeah I am not sure.

Maybe distractions, like for me what was good like I said was how I was able to go elsewhere. I was lying flat so I couldn’t see what was going on. I could just be distracted by myself. Obviously, they can’t do anything with their hands but maybe there is a TV in there or something they can go elsewhere with while it is happening. But the process I was very comfortable with.

Maybe have some jellybeans.

I mean if you doing it for kids I would imagine. I mean if you don’t do it already, parents being present or running distractors, is generally a good idea. When I started utilising my own distractors that made the experience much more bearable.

Or perhaps some sort of entertain playing for them to focus on. Because I found that taking my mind of the pain helped a lot with the process.

One of the things that I was thinking to mention to you was that maybe if you do it to children having them laying down like I was but to have something distracting them, looking at, up you know on the ceiling or something like that I don’t know. But they could be distracted from that and I think that that would go nicely.

It could be music, it could be anything really. But I think that the key on that will be the doctor or the nurse that is doing the procedure. The way that they would come across I think is very important and I feel that sometimes when a doctor doesn’t treat me well or doesn’t, it like in a negative way. It is very important I think. That is what you guys achieved. To make me feel comfortable, to make me feel safe particularly. I am a grown guy so, but I am seeing from the perspective of a child. Children want to feel safe and they want to feel that. They don’t think about it, they just feel it. I think that is very important the way it is going to be applied and with whom, and the kind of environment that they are going to be surrounded by, it has got to be comfortable because the pain that I felt, that I could manage is not necessarily comfortable for a child.

May be have headsets for the kids, or some kind of like TV. If they felt anxiety you can let them have headphones on and some kind of music or something. The scale of the light, because sometimes the lights can be scary if they close your eyes or something. Sometimes like blankets because it makes them feel like cosy, comforting.

## Provision of additional analgesia during and after the infusion

Despite a local anaesthetic being provided, many participants felt that it was inadequate for a paediatric cohort and additional pain relief would be likely if the procedure was to be undertaken on a child.

I think that is good enough. I don’t know of the pain that people or kids feel could be a bit more of anaesthetic or something like that, but for me it was all right

Interviewer: More anaesthesia?

Response: Yeah, especially if it is for kids. I don’t have the biggest pain tolerance but I don’t think kids would have more of a pain tolerance than I do.

I guess more anaesthetics is not a good thing, but definitely because I can see them having like less of a threshold for pain tolerance. I guess the explaining of what the doctor is doing the whole time is like comforting in a sense so that was good. Yeah, and the counting. The counting for me really helps ever since I was a kid that was what I was like used to. Like 1 2 3.

I don’t know if much more local anaesthesia would be a good idea, like I imagine there is some factors that play in it. Is anaesthesia something that just wouldn’t go well with the injection? Like it is one of those drugs?

Furthermore, some participants noted that additional pain relief in the days following the infusion may allow for less side effects and pain as they experienced, particularly due to the active lifestyle of most kids. This also included therapies like hot or cold compresses around the area.

Maybe I didn’t use cold compress or warm compress or something to reduce the swollenness or the redness or whatever. In my work, for in my case, I just wait and we will see how it happened but everything went well. I just recovered day by day. I am feeling better and better. My skin is looking better as well.

I guess, it would depend on the children’s lifestyle. If they would be doing a lot of bending over maybe before their play or sport or what not, but I don’t think it would be much of a problem which site really they get injection into.

Just going back to the children and feeling discomfort about the bulge thing. They are going to be very active as well so, and it took about a week for this thing to decrease. So I don’t know if children are able to maybe wait a couple of days until the thing has calmed down a bit before they get more active because I know from my experience that if restrict a child who is too much active, you know they get very reactive and can get a bit out of hand so I think that might be a good thing to think about when it comes to like maybe children’s activities and restricting their own freedom when it comes to letting it settle down before they are able to move much more freely.

Yeah, it would be helpful to tell them like if you don’t want to feel more pain tomorrow it is better to take today in a relaxed way. Don’t do exercise, relax, stay in bed. Do as little as you can.

I can see that, especially with young kids, it would be harder for them because of dealing with the pain. But they gave the anaesthetic which obviously helps a lot more than I realised when I got to the next day, I realised that the anaesthetic was actually doing more than I realised. Maybe for the next day if you had some form of painkillers that would work but I don’t know because I wasn’t allowed to take any of those, I don’t think. I don’t know.

But I regret not doing it and I think if you guys are going to do this with kids, whether they are in pain or not to just give them a little paracetamol or something like that just to fall back on instead of getting them to say I am in pain, to avoid that I think that. I regret not taking it because you guys offer it to me.

## Slowing down the infusion time

While this theme was somewhat explored above, the below quotes are associated specifically with why slowing the infusion would be beneficial for children.

Reduce the redness and swollenness or it could be painful for them but if you infuse slower it could be better for the kids.

Things like that, it was less painful I guess in other words. I think that I guess like slower infusion time. Does that make sense?

I was thinking about that maybe you should try, I guess, a slower, sorry a much longer dose time. So it goes into the body slowly and maybe there are some distractions that can be used to distract the children while they are being injected and, I guess looking back at my own experience with this bit of a bulge for my day to day I guess. It may depend if the children has, it is probably a bad thing to say, but if they have a high percentage of fat that could feel less pain compared to those who are very lean I guess. But I don’t know if this dosage is like the proper amount or maybe it can be decreased a little bit just so you can improve that pain after being injected.

I would say maybe decreasing it would be a better idea but then again, I am not quite sure if they will improve the children’s experience with it. Because I imagine those who are much leaner than me with that much amount in their stomach will definitely have a, you know, much harder time to live day to day.

Slightly longer, yeah, and then it would be less painful.

Well assuming a faster injection would be causing more pain, definitely a slower one would help. It would probably help to have the parents in the vicinity to keep the child company.

## Ensuring information is appropriate for a child to understand and consent to

Four participants highlighted the need to ensure the information delivered to young participants or patients prior to the infusion commencing adequately prepared them for the experience ahead. While this is a given with any paediatric study, helpful suggestions are noted below.

There would have to be some kind of fun to it I guess. The explanation to me was fine but I don’t know who you would explain it to a kid. Dumb it down or word it differently. But it all made sense to me anyway.

I guess as much information as humanly possible at that kind of level and I think pictures would help as well. I don’t have children myself but I think that children are very visual people so I think understanding, I don’t know how you would do it, by either in a cartoon kind of thing maybe, or posters like a comic version, something that would catch their imagination of. I remember as a kid going to the doctors and dentists and stuff, and there would be cartoon teeth doing something. I don’t know if you can do it that way, making it visual and them understanding what is happening and why there is a needle that has to go into them and why it is going to hurt and why it going to get hard. You know, those kind of things. So when it happens it is not a shock I guess. So the more information without saying dummy it down for a kid but making in the language I guess and in a style that a kid is used to rather than medical terms and abbreviations or things like that.

Obviously the manner in which you would approach a kid would be different. I can’t think of anything other than that. Like the same way you interacted with me wouldn’t be the same as you would interact with like a younger kid.

I will say which we discussed just now was just to explain to them what this process will be, the actual dosing and definitely make them aware of the pain, which of course, I know that you are going to do that, hence, all the questions you have been asking. Socially, yeah, because of that ages let them know. But I think at the end the people will bounce back fast.

## Using numbing cream prior to inserting the catheter

Despite most participants having a positive and mostly painless experience with the insertion of the abdominal catheter, reflections upon how children would perceive that procedure elicited suggestions regarding numbing the area prior to insertion. This was specifically to avoid the ‘stinging’ sensation associated with the insertion of the local anaesthetic.

Yeah, but maybe for some kids you need to put the numbing cream in that area first. The numbing cream maybe you have to numb the skin. So when the first initial sharp scratch they might not feel it with the numbing cream.

I mean if there is some way to numb the area a bit more for the kids that might be a better way maybe.

That was just the painful bit, just putting the needle in, so I don’t know if there is some way to numb the area first.

I actually felt a bit of pain from the actual needle going in so I don’t know if there is any way to numb the area first.

## Using two infusion pumps as opposed to one

Two participants – who both had children and were therefore able to reflect on these questions slightly deeper – stated that using two pumps instead of one might be preferable. Both experienced little pain during the initial half and a large amount as the infusion finished. Given neither felt the insertion of the catheter, they perceived this as an avenue worth exploring.

Like if you give two injection pumps at the same time, halving the dose because it only hurt going this way, like it didn’t hurt this way at all…

but like at the same time so they are not having to come back. But if you can get two needles into a kid somehow. Because to be honest the needle didn’t hurt at all.

I think being hooked up for fifteen minutes is quite daunting. If you told a kid you are going to be sat there with a needle in you and getting injected for fifteen minutes, I think they would be a bit scared. I don’t know if that is the way I have taken in like in two parts because obviously the first part of the process is quite easy, towards the end like when you getting into, you have passed your ten minute mark it starts getting a bit like in your face.

# The pain experienced during the infusion was likely too intense for a child

Eight participants expressed sentiments that the infusion process in its current form was unacceptable for children and would likely not be tolerated due to the pain experienced. This was particularly relevant given participants were aware the process was to be completed every three months for those who required secondary prophylaxis, perceiving pain to be a factor that affected continued treatment. At present, this is a current issue with the standard therapy.

My pain factor is pretty high so for a child that probably would hurt quite a lot.

I think maybe this dose would definitely work on children who have a higher fat percentage than others but for some leaner children I would say to reduce it by a little bit.

Maybe like pain management because I could see kids having an issue with that harsh stinging pain at the end. I think that is maybe the only thing that maybe look into.

I don’t think a kid would like to go through that kind of pain because if a kid goes through that kind of pain, because I know the injection is meant to be ????, they have that pain and then they are told to come back to have the same dosage again, they would be quite reluctant to come back.

Interviewer: If you think of your kids, would they be tolerable with this amount of pain?

Response: No. Like the needle yeah, the anaesthetic maybe, but that second pain no. They wouldn’t even stay still for it.

If I was a kid I mean I would be in pain but if I was just on Nurofen and Panadol I would probably be fine. But I can imagine, you know, if got a particularly sensitive child it might be a bit painful but I feel like it would be better than the arm because then you would actually not to be able to move your around.

I am managing so far, like it hurting a little bit more right now. I can’t imagine like what is a way we could to talk about kids, like me six years old getting this, I would be so mad.

I would imagine, it would be pretty hard to convince them about the pain. Like for younger children once they get a bit of pain they will be like screaming at the pain, but it is just depending on them, the individual I guess. Some may be more reasonable than others. But adults they would probably need. They would understand the need for something like this because the first line would be oral antibiotics. The infusion to be used only if absolutely necessary.

# Overall experience with the trail

It was promising that despite the pain many experienced, most participants had a positive experience with the SCIP Trial, as substantiated below.

## A positive trial experience

Noting that these quotes have not been compared against pain scores or additional data to determine if those who had a positive trial experience had markedly less pain than other participants, these data are promising.

Everything went smooth and good, I guess, basically. Everything as expected, or as I expected.

I think all the procedures went well, in my opinion, my experience more than my opinion. I didn’t feel pain at all so it is fine.

My experience so far has been fine. I am not traumatised or anything. So everything is okay.

I think, with the way the research is going I am quite adamant that this is going to be positive what comes out of it.

No issues. From the screening all the way through.

It was not too bad. It has been pretty easy going. It is not hard to go through.

It wasn’t too bad. From recruitment to actually doing the thing, it was only one or two weeks I think. The only thing is that the drive here is quite long and then parking but other than that it has been fine yeah.

I felt no pain during it and the process was fine. I felt comfortable.

Just thank you for making this process as it has been for my first clinical trial. It has been a pleasure.

It was good to know what was happening, interesting.

I guess the way everything like the infusion itself was managed. You frequently get asked questions, you know, pain levels, if we need to slow down the rate during the infusion or anything like that.

The process has been very, very smooth. I have no regrets about doing this.

It has been okay. It was done very well. The process was efficient and effective.

It was effective and everyone was very professional. I cannot fault the process. If someone can then maybe something happened to them individually but even socialising with my fellow participants, no one said anything negative.

## Participants felt supported by the team and had positive interactions

As a member of the trial team present for two of the five dosing days, the following quotes are very heartening and it is positive that the team were held in such high regard by participants.

It has been interesting. In that I haven’t done it before, so just experienced enough support and well I think expected procedures and I am feeling good, like physically good and supported by the team.

The interactions were fine. You guys told me what you were doing and teaching like the two nurses and like showing them what was happening with scan. Yeah, it was fine.

I think personally for me it was just the anaesthesia bit, like you guys interacting with me was all good. You guys were very friendly.

I think that it was okay. Nerves and anxiety got to me during the actual dosing day but throughout the whole, like check-ups, ever since then has been like really nice and everyone has been really supportive and really kind to me.

I think for like all of the people involved in it were very supportive and like very caring and made me feel as comfortable as I could feel at the time I think. Like everyone has just been like super nice so it made me look forward to going to check ups and even during dosing everyone just made me feel as comfortable as I could feel because why make someone feel more anxious when they are stuck in a hospital bed.

You guys are very professional.

I think the way you guys did it, it helps the procedure to be easier for the client, the patient. Explaining everything. As I told you, being able to see what is going on, it really eased my mind. The security of Rob’s talk was very nice. It helps a lot to relax.

No, I think you guys have been really professional. It has been really interesting process and friendly and stuff. That helps a lot to feel like those guys know what they are doing.

It seemed very safe to me but the doctor that did it and the health team and yourself, the way you guys work as a team is pretty good. I was very comfortable. Very comfortable with the three of you that were there. The doctor that operated the scanner, you know, when nice people like yourselves are trying to team up and I am there, I am the subject, I am happy.

I think that is very important to somebody who participates, particularly while the process was going on. You were very helpful. That was very good.

It has been really good. All the staff were friendly and they take care of you so it has been a pretty good experience.

So far it has been done effectively, efficiently, professionally. I can’t fault your process. If I missed something I apologise.

## The trial was well organised and suited participants other commitments

Given the extended nature of the trial’s follow-up appointments and the length of dosing day, it was promising that participants expressed that they were able to combine their trial responsibilities with other commitments and were enabled autonomy in their decision to stay in confinement for the evening.

The sleeping worked really well, having been allowed to stay on the ward afterwards that was really convenient, and then we just walked just over for the outpatients the next day. That was really handy, I think, had I have had to been discharged at night and then come back, I have got a forty minute drive each way, so it probably would have been quite challenging. But that was really convenient. I was pretty happy with that

I am pretty happy and I know what is expected and I have got my appointments planned, so I am pretty happy.

No, I think everything is fine for my experience. I think everything went well and I feel well too. Nothing big.

I guess everything leading up to the injection and everything after that as well. Ultimately, I think, it has all been pretty well even when I had to, you know, change some times. You guys know pretty well with managing that so. It worked pretty well.

Yeah, good. The hospital stay was really good and with the doctor.

I think you are all doing your job wonderfully.

it has been a very easy process, very straightforward process and the only barriers I see for people are the frequent injections and needles and what not, but that stuff that needs to happen so I can’t really suggest any thing I can see being done different that doesn’t need to happen.

It was a good preparation. That was important to make everything go smooth.

I did like the fact that everything was done, organised properly, the whole thing I was expecting that.

## Information was provided often and questions answered

Participants expressed feeling able to ask questions as often as required while receiving answers in a timely and easy to understand manner.

I think you have kept me updated all the way through. The team have been pretty good.

I was always asked questions, how I am feeling and doing. All their recommendations that you usually do in these kind of processes or trials. Always looking after me.

Yeah, yeah. I understand all the process, how it going to be and how I am going to feel, like if I am going to feel something or expected symptoms after the infusion.

As I told you, everything went well. The team told me things that I might experience so everything went well to be honest.

This is quite informative because the team, like you, you were one of the team members, I had so many questions you answered patiently. When I had so many fears you answered quite patiently and this is quite good, quite good.

The doctor that explained to me, she explained in a straightforward way

Very easy, very straightforward. I was able to understand everything required of me and everything that I needed to do.

## Likelihood of future participation

Two participants stated outright that they would not participate in the trial again if given the opportunity, while two said they would. Reasons for this are supported with quotes below. Notably painful infusions were experienced by those who would not return,

I wouldn’t do it again of my own free will. If I had to obviously get it done then I would but I would not that trial again. If that makes sense because it was painful after the ten minutes, and then it was like a little bit for the first two days as well it was a bit sore. I wouldn’t do the trial again…I probably wouldn’t, even if I got paid for it.

Like the process and stuff and the fact that you are sat here watching things happening. It is quite a long hour dragged out process.

Painful would probably be a good word to use. Just because you don’t take into account how far way in the appointment. You kind of know the appointments are after but just like the parking side of it, the travel side of it has been difficult for me anyway. Then obviously because you are trying to book rooms and stuff it has moved the Fridays around a lot which has affected my work and stuff. That probably has only really been the biggest drama for me I would say.

I would do the infusion again, yes, most definitely. Just to go back to the question about delivering to teenagers. My eldest is ten years old if he had the problem I would happily tell him to get it.

Interviewer: Would you do it again, if I said I would put it in deeper, would you do it again in say like three- or four-months’ time?

Response: yeah. (inaudible) I do trust you so I absolutely yeah I will yeah.
