# Supplementary material for: Qualitative assessment of healthy volunteer experience receiving subcutaneous infusions of high-dose benzathine penicillin G (SCIP) provides insights into design of late phase clinical studies
Source: PLoS One. 2023 Apr 27;18(4):e0285037. doi: 10.1371/journal.pone.0285037 (PMC10138475; doi:10.1371/journal.pone.0285037)
Supplement: Attachment — Submitted filename: Response to Reviewers.pdf [file pone.0285037.s002.pdf]

TELETHON KIDS INSTITUTE

Northern Entrance
Perth Children's Hospital
15 Hospital Avenue
Nedlands WA 6009

PO Box 855,
West Perth WA 6872

ABN 86 009 278 755

T | 61 8 6319 1000
E | info@telethonkids.org.au
W | telethonkids.org.au

Proudly supported by the people of Western Australia through Channel 7's Telethon

13th March 2023

Dear Dr. Arteaga-Livias

Thank you for the opportunity to submit a revised version of the original research article entitled "Qualitative assessment of healthy volunteer experience receiving subcutaneous infusions of high-dose benzathine penicillin G (SCIP) provides insights into design of late phase clinical studies" (PONE-D-22-31775).

We are grateful for the time taken by the reviewers to improve this piece of work and as such have made the following changes.

1. *(Editor): Please ensure that your manuscript meets PLOS ONE's style requirements, including those for file naming.*

The manuscript has been amended to meet PLOS ONE style requirements.

2. *(Editor): Please provide additional details regarding participant consent.*

All participants provided written, informed consent. Additional information to clarify how consent was obtained and what types has been included in the manuscript and online submission statement.

3. *In the ethics statement in the Methods and online submission information, please ensure that you have specified (1) whether consent was informed and (2) what type you obtained (for instance, written or verbal, and if verbal, how it was documented and witnessed).*

All participants provided written, informed consent. This has been included in the Methods of the manuscript and the online submission information.

4. *(Editor): Thank you for stating the financial disclosure: Please state what role the funders took in the study. If the funders had no role, please state: "The funders had no role in study design, data collection and analysis, decision to publish, or preparation of the manuscript."*

These statements have been provided accordingly.

5. *(Editor): In your Data Availability statement, you have not specified where the minimal data set underlying the results described in your manuscript can be found. For more information about our data policy, please see http://journals.plos.org/plosone/s/data-availability.*

A supplementary file with a minimal data set has been provided.

6. *(Reviewer 1): 'Many of the findings revolve around specifics of infusion with respect to speed and anesthesia. It would be interesting to know if the investigators are motivated by these results to define alternative infusion protocols when adverse symptoms are experienced.'*

We thank reviewer 1 for this suggestion and as this is being explored in the second phase of the research (SCIP-II) have included a brief notation as to considerations of strategies.

7. *(Reviewer 1): Missing from the interviews was a question along the lines of "If you felt it was medically important, would you be willing to attend every three months for a repeat infusion." One infusion may not indicate long term tolerability. It will be interesting as the underlying clinical trial proceeds to ask patients who have previously been receiving monthly injections how they perceive the subcutaneous infusion as an alternative.*

As the next iteration of the study is enrolling participants with ARF and RHD who are presently undergoing monthly injections, this question is being asked to serve as a point of comparison. Further information to this point is included in the discussion.

8. *(Reviewer 1): Correct this sentence: "In the week following the SCIP, there was no suggestion the procedure did not appear to disrupt the lives of the participants…"*

This sentence has been amended to read as follows: 'In the seven days following the infusion, there was no indication that the procedure disrupted the lives of the participants.'

9. *(Reviewer 2): Specify whether infusions occur by gravity or via a pump.*

Amendments have been made to the methods to clarify that the infusion occurred via a a spring-driven syringe infusion pump (Springfusor®30, Go Medical Industries Pty Ltd., Subiaco, Australia), a variable flow control device (VersaRate® Plus, EMED Technologies, El Dorado Hills, California, USA) and a 22G SC catheter (BD Saf-T-Intima™, BD Medical, Mississauga, Ontario, Canada).

10. *(Reviewer 2): Were interviews only conducted face-to-face or via telephone as well?*

All interviews occurred face-to-face and this has been clarified in the methodology.

11. *(Reviewer 2): Not withstanding that this is a Phase I study, there are references for but limited discussion of potential issues for introducing this to the intended target population. Some further discussion around difficulties and culture-appropriate techniques for engagement and clear communication with a remote Indigenous population (children, youth and adults) in view of these results would be helpful.*

We thank Reviewer 2 for this comment and note it to be an important consideration. A further paragraph has been included in the discussion specific to this point.

All authors continue to have no conflicts of interest to disclose. The funders had no role in study design, data collection and analysis, decision to publish, or preparation of the manuscript.

Please address all correspondence concerning to myself at stephanie.enkel@telethonkids.org.au. Thank you for your consideration of this revised manuscript.

Sincerely,

Miss Stephanie Enkel
On behalf of all authors.
